# Evolutionary Conservation of the Copper-Dependent Thiol Oxidase Activity of Selenium-Binding Proteins

**DOI:** 10.3390/antiox15091148

**Published:** 2026-09-10

**Authors:** Hanna Schlemminger, Swantje Melina Lockowandt, Alina Löser, Anna Patricia Kipp, Lars-Oliver Klotz, Holger Steinbrenner

**Affiliations:** 1 Institute of Nutritional Sciences, Nutrigenomics Section, Friedrich Schiller University Jena, D-07743 Jena, Germany; hanna.schlemminger@uni-jena.de (H.S.); swantje.melina.lockowandt@uni-jena.de (S.M.L.); 2 Institute of Nutritional Sciences, Department of Nutritional Physiology, Friedrich Schiller University Jena, D-07743 Jena, Germany; alina.loeser@uni-jena.de (A.L.); anna.kipp@uni-jena.de (A.P.K.)

**Keywords:** MTO, SELENBP1, methanethiol, H_2_S, volatile sulfur compound, copper, selenium, evolution

## Abstract

Human selenium-binding protein 1 (SELENBP1) is a methanethiol oxidase (MTO), converting methanethiol (MT) to hydrogen sulfide (H_2_S), hydrogen peroxide (H_2_O_2_) and formaldehyde (HCHO). SELENBP1 has orthologs in all domains of life. RdMTO, an orthologous enzyme recently identified in the marine bacterium *Roseobacter denitrificans*, was postulated to require the cysteine residue closest to its C-terminus, Cys448, for MT binding and to oxidize MT to sulfane sulfur (S^0^) rather than to H_2_S. SELENBP1 and RdMTO exhibit ~53% sequence identity, with Cys448 (numbered Cys466 in SELENBP1) and amino acids required for copper binding being conserved. Therefore, we here compared the MTO activity of recombinant SELENBP1 and RdMTO. We found that SELENBP1, like RdMTO, converts MT as well as structurally related alkyl thiols to form H_2_S, H_2_O_2_ and, in the case of MT, HCHO in a strictly copper-dependent manner. MTO activity of both proteins was lowered but not abrogated upon mutation of their respective C-terminal cysteine residue. Thus, the catalytic mechanism of selenium-binding proteins that act as copper-dependent MTOs appears to be evolutionarily conserved from bacteria to humans. Presumably, this enzyme is an early evolutionary invention of prokaryotes, in order to cope with toxic thiols in their environment.

## 1. Introduction

Human selenium-binding protein 1 (SELENBP1) was first described in 1997, following the identification of its murine ortholog through ^75^Se-radiolabeling of liver proteins [1,2]. SELENBP1 has been implicated in a multitude of physiological processes such as protein transport and degradation, lipid metabolism, redox homeostasis, cellular proliferation and differentiation. Moreover, it is considered to be a tumor suppressor [3,4,5,6,7,8,9,10]. An enzymatic function of SELENBP1 as a methanethiol oxidase (MTO) was discovered in 2018: SELENBP1 catalyzes the conversion of a toxic volatile sulfur compound (VSC), methanethiol (MT), to hydrogen sulfide (H_2_S), hydrogen peroxide (H_2_O_2_) and formaldehyde (HCHO) [11]. Single nucleotide polymorphisms (SNPs) in the *SELENBP1* gene, resulting in diminished MTO activity, have been associated with extraoral halitosis, characterized by an unpleasant cabbage-like breath odor [11]. Downregulation of SELENBP1 in tumor tissue may contribute to elevated levels of MT and its methylated derivatives in body fluids, breath and/or flatus of cancer patients [7].

Phylogenetic profiling revealed the presence of SELENBP1 orthologs within the proteomes of more than 1000 species, including vertebrates, invertebrates, plants, bacteria and archaea [12]. MTO activity was demonstrated for the orthologous proteins in *Caenorhabditis elegans* (*C. elegans*) as well as in malodorous plants and some bacteria [13,14,15,16,17]. We have recently shown that human SELENBP1 and a SELENBP1 ortholog with MTO activity (SEMO-1) from *C. elegans* are copper-dependent enzymes [18]. In addition to MT, SELENBP1 accepts several structurally related alkyl thiols as substrates for oxidation [18]. The amino acids His137, His140, Asp189 and Glu252 in SELENBP1 (conserved as His135, His138, Asp187 and Glu252 in SEMO-1) were identified to be critical for both copper binding and MTO activity [18]. Like SELENBP1 and SEMO-1, a bacterial MTO from *Hyphomicrobium* sp. requires copper as a cofactor for enzymatic activity [14].

MT represents a key intermediate in the global biogeochemical sulfur cycle. MT-producing and -degrading microorganisms flourish in diverse environments, including terrestrial and marine ecosystems as well as the gastrointestinal tract of mammals [19,20]. In humans, MT is primarily produced by colonic microbiota through degradation of methionine, a sulfur-containing amino acid derived from dietary proteins [7]. Microorganisms in seawater also generate high amounts of MT, mainly through demethylation of dimethylsulfoniopropionate (DMSP), an osmolyte abundantly present in phytoplankton [20,21,22]. For both humans and microorganisms, MTOs provide a means for MT detoxification as well as for the production of metabolites that may serve as carbon and sulfur sources. In this regard, Cao et al. recently identified 57 putative MTOs in genomes of the *Rhodobacteraceae* family, including RdMTO in the marine bacterium *Roseobacter denitrificans* (*R. denitrificans*), which they picked as a representative enzyme for further investigation [13]. Their findings suggest the formation of per-/polysulfides as intermediates of sulfane sulfur (S^0^), resulting in S^0^, HCHO and H_2_O as postulated final products of MT oxidation, rather than H_2_S, H_2_O_2_ and HCHO. In addition, Cys448, the cysteine residue closest to the C-terminus of RdMTO, was postulated to mediate substrate binding [13]. Interestingly, Cys448 is conserved (as Cys466) in human SELENBP1.

In this study, we compared the MTO activity of recombinant human SELENBP1 and RdMTO, with emphasis on the role of copper as cofactor and on the role of the conserved C-terminal cysteine residue. Following the observation that key amino acid residues required for copper binding in SELENBP1 and SEMO-1 are also conserved in RdMTO, we demonstrate the capability of RdMTO to convert MT and two structurally related alkyl thiols to H_2_S, H_2_O_2_ and, in the case of MT, HCHO, in a strictly copper-dependent manner. The C-terminal cysteine residue was indeed found to be important, yet not essential for copper-dependent MTO activity of both SELENBP1 and RdMTO. This suggests evolutionary conservation of the catalytic mechanism of MTOs from bacteria to humans.

## 2. Materials and Methods

### 2.1. Reagents

Chemicals were purchased from Carl Roth (Karlsruhe, Germany) or Sigma-Aldrich/Merck (Darmstadt, Germany), if not stated otherwise. Primers were obtained from Life Technologies (Darmstadt, Germany).

### 2.2. Alignment of Protein Sequences and Phylogenetic Analysis

Protein sequences were obtained from the UniProt database (https://www.uniprot.org/). Analysis of amino acid conservation and alignment of sequences between human SELENBP1 (Q13228) and RdMTO (Q166Z5) was performed using the program Jalview, as available at www.jalview.org, using the Clustal Omega approach with default settings, as previously described [23].

A phylogenetic tree was compiled of MTOs from diverse organisms, using the Clustal Omega Multiple Sequence Alignment (MSA) tool (https://www.ebi.ac.uk/jdispatcher/msa/clustalo?outfmt=fa (accessed on 7 September 2026)) with default settings, as provided by EMBL-EBI [24].

### 2.3. Plasmids for Protein Overexpression in Escherichia coli (E. coli)

Expression plasmids for bacterial production of recombinant Strep-tagged methionine γ-lyase (MGL) and human SELENBP1 were previously introduced [25]. The sequence encoding RdMTO was obtained from the Kyoto Encyclopedia of Genomes and Genes (KEGG) database (https://www.genome.jp/kegg/ (accessed on 7 September 2026)). It was amplified by PCR from genomic DNA of *R. denitrificans* OCh114, purchased from the German Collection of Microorganisms and Cell Cultures (Leibniz Institute DSMZ, Braunschweig, Germany). Q5 high fidelity DNA polymerase (New England Biolabs, Ipswich, MA, USA) and the CP1 primers specified below were used for this first PCR. Thereafter, a second PCR was performed, using the purified product of the first PCR as a template and the CP2 primers. The purified product of this second PCR was digested with NdeI and Eco47III and subsequently ligated into the SELENBP1 expression plasmid prepared with the same restriction enzymes, thereby replacing the sequence encoding SELENBP1. Sequences of the primers used for cloning are listed in Table 1.

The plasmids for production of SELENBP1 Cys466Ser and RdMTO Cys448Ser mutant proteins were generated by site-directed in vitro mutagenesis based on PCR as described [25]. Sequences of primers used for mutagenesis are listed in Table 2, with exchanged bases marked in bold and underlined.

### 2.4. Bacterial Overexpression and Purification of Recombinant Proteins

Production and isolation of recombinant Strep-tagged SELENBP1, RdMTO and MGL was carried out as described before [18,25]. Briefly, *Escherichia coli* (*E. coli*) KRX bacteria were grown in Luria/Miller LB media supplemented with 50 µg/mL carbenicillin at 37 °C, and biosynthesis of recombinant proteins was induced by adding L-rhamnose (Carbolution, St. Ingbert, Germany). If applicable, CuCl_2_ dissolved in HEPES-buffered saline (HBS) (50 mM HEPES, 150 mM NaCl, pH 7.4) was supplemented to a final concentration of 150 µM upon induction. Temperature was then lowered to 22 °C, and bacteria were incubated for 3 h at 200 rpm. Bacteria were harvested by centrifugation, and pellets were washed once with HBS, resuspended in HBS and lysed by sonification. The soluble fraction was separated by centrifugation and transferred to a Strep-Tactin^®^ 4Flow^®^ column (IBA Lifesciences, Göttingen, Germany) that had been prewashed with HBS and 10 mM NaOH. Contaminating proteins were removed by successive washings with HBS, chaperonin buffer (20 mM MgCl_2_, 5 mM ATP in HBS) and HBS. Elution of the recombinant proteins occurred with 50 mM biotin (IBA Lifesciences) dissolved in HBS. Buffer change was accomplished by ultrafiltration using Vivaspin 6 centrifugal concentrators with a Molecular Weight Cut Off (MWCO) of 10 kDa (Sartorius, Göttingen, Germany). Aliquots of the proteins were stored at −80 °C.

### 2.5. SDS-PAGE and Immunoblotting

Protein concentrations were determined using the Pierce™ BCA Protein Assay Kit (Thermo Fisher Scientific, Waltham, MA, USA). SDS-PAGE and blotting to PVDF membranes (Carl Roth) were carried out following standard procedures as described before [18,25]. Protein purity was verified using a standard protocol for Coomassie staining of SDS-PAGE gels. After blocking, membranes were probed with an anti-Strep-tag antibody (mouse StrepMAB-Classic, IBA Lifesciences), followed by incubation with a horseradish peroxidase (HRP)-coupled antibody (goat anti-mouse IgG, Thermo Fisher Scientific) and SuperSignal West Pico Substrate (Thermo Fisher Scientific). For detection and analysis, a ChemiDoc™ MP analyzer equipped with Image Lab 6.1 software (Bio-Rad Laboratories, Munich, Germany) was used.

### 2.6. Determination of Thiol Oxidase Activity

MTO activity was explored by determining the three products of MT oxidation: H_2_S, H_2_O_2_ and HCHO. MT-derived H_2_S and H_2_O_2_ were detected by a coupled assay as described before [18,25]. Briefly, 10 µg of recombinant SELENBP1 or RdMTO were applied as duplicates in adjacent wells of a transparent 384-well plate (Greiner Bio-One, Frickenhausen, Germany). Where applicable, metal cations were supplemented as chloride salts dissolved in HBS to a final concentration of 15.38 µM (1:2 protein/cation), and the reaction mix was made up to a final volume of 40 µL with HBS; 20 mM L-methionine dissolved in HBS were pipetted into an adjacent well. After adding 0.225 mg/mL MGL (or HBS for the negative control) to the methionine solution, the plate was immediately covered with a filter paper soaked in 20 mM lead acetate and sealed with adhesive tape. The plate was incubated for 3 h at 37 °C and 150 rpm. Detection of PbS spots derived from the reaction of volatile H_2_S with lead acetate was conducted using the ChemiDoc™ MP analyzer. For determination of H_2_O_2_, the Amplex^TM^ Red Hydrogen Peroxide/Peroxidase Assay Kit (Thermo Fisher Scientific) was applied, measuring fluorescence (λ_ex_: 571 nm, λ_em_: 585 nm) in black 384-well plates (Greiner Bio-One) in a CLARIOstar plate reader (BMG Labtech, Ortenberg, Germany). For determination of HCHO, 68.4 mM Purpald^®^ reagent dissolved in 1 M NaOH was added to the MTO reaction mixes. After incubation for 15 min, absorption was measured at 549 nm using a SPECTROstar Nano plate reader (BMG Labtech).

In addition to MT, ethanethiol (ET) and propanethiol (PT) were tested as potential substrates of RdMTO. ET was enzymatically generated through MGL-catalyzed degradation of its precursor amino acid ethionine (20 mM DL-ethionine in HBS), while PT was added directly to the MTO reaction mix at a final concentration of 100 µM [18].

### 2.7. Analysis of Copper Content

Copper content of purified recombinant proteins was determined by total reflection X-ray fluorescence (TXRF) spectrometry, using a bench-top TXRF spectrometer (S4 T-STAR, Bruker Nano, Berlin, Germany) and 1 mg/L Yttrium (Merck/Millipore, Darmstadt, Germany) as internal standard, as described before [18].

### 2.8. Computational Prediction of Disulfide Bonds

Three-dimensional (3D) structures of SELENBP1 (UniProt ID: Q13228) and RdMTO (UniProt ID: Q166Z5) were extracted from the AlphaFold database (https://alphafold.com/). Prediction of disulfide bridges via measurement of the spatial distance between thiol groups of cysteine residues was performed using PyMOL version 3.0 (PyMOL Molecular Graphics System by Schrödinger, DeLano Scientific LLC, San Francisco, CA, USA).

### 2.9. Statistical Analysis

Data are depicted as means ± SD, unless stated otherwise. For statistical analysis, Student’s *t*-test or one-way ANOVA with Dunnett’s multiple comparisons test was used, as indicated; *p* < 0.05 was considered as statistically significant. All calculations were performed using GraphPad PRISM, version 8.0.1 (GraphPad Software, San Diego, CA, USA).

## 3. Results and Discussion

### 3.1. RdMTO and SELENBP1 Are Orthologous Proteins Sharing Conserved Amino Acid Residues and Sequence Motifs

**Figure 1:**
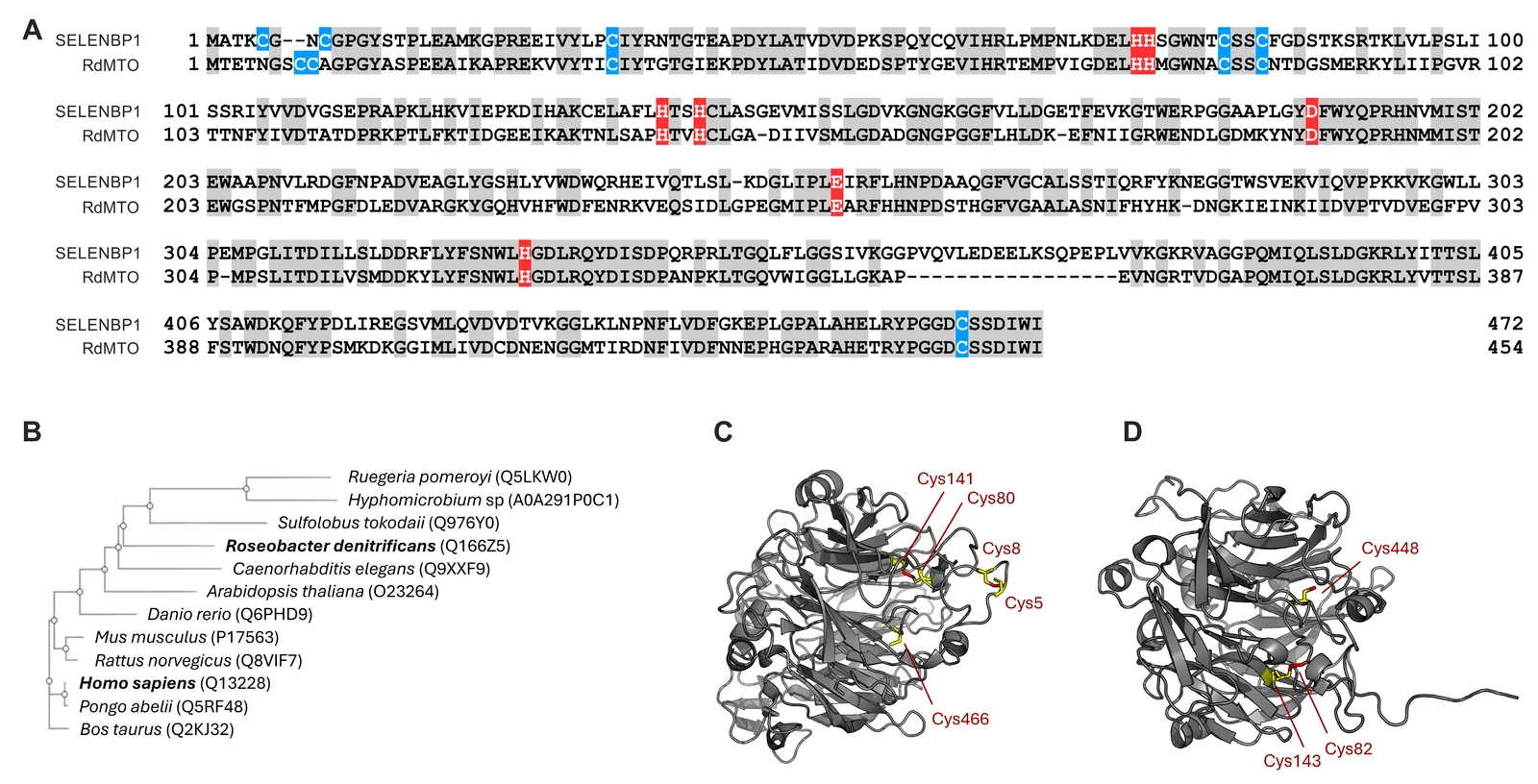
Comparison of human SELENBP1 and RdMTO. (**A**) Pairwise alignment of SELENBP1 (UniProt ID: Q13228) and RdMTO (UniProt ID: Q166Z5) with conserved cysteine residues framed in blue and conserved amino acid residues putatively linked to copper coordination framed in red. Other amino acids that are identical in SELENBP1 and RdMTO are shaded in grey. (**B**) A phylogenetic tree of orthologous SeBPs/MTOs from diverse species. The UniProt IDs of the proteins are indicated in brackets after the respective organism. 3D protein structures of SELENBP1 (**C**) and RdMTO (**D**) were obtained from the AlphaFold 3 database. Selected cysteine residues are highlighted in yellow, with potential disulfide bonds (distance between thiol groups ≤ 2.5 Å) shown in red. Calculations of the spatial distances and prediction of disulfide bonds were carried out using PyMOL.

Screening the genomes of *Rhodobacteraceae* for orthologs of the gene encoding an MTO present in the marine bacterium *Roseobacter pomeroyi*, Cao et al. recently identified RdMTO from *R. denitrificans* as a novel member of the selenium-binding protein (SeBP) family [13]. In order to assess similarities with SELENBP1 from *Homo sapiens*, we first performed pairwise alignment of the protein sequences of RdMTO (UniProt ID: Q166Z5) and human SELENBP1 (UniProt ID: Q13228) (Figure 1A), revealing an overall sequence identity of ~53% with numerous conserved amino acid residues: seven out of the 10 cysteine residues in SELENBP1 have corresponding cysteine residues in RdMTO, including Cys5/8 and Cys80 (Cys8/9 and Cys82 in RdMTO) that were previously linked to selenium binding by SELENBP1 [18]. Additionally, a cysteine-containing CSSC thioredoxin motif starts with Cys80 (Cys82 in RdMTO). The 13 C-terminal amino acids of the two proteins are identical, including the cysteine residue closest to their C-terminus (Cys466 in SELENBP1 and Cys448 in RdMTO, respectively) that was recently proposed to be indispensable for the enzymatic activity of RdMTO [13]. Moreover, both proteins possess two metal-binding motifs as well as the six amino acid residues (His73/74, His137/140, Asp189, Glu252 in SELENBP1, conserved as His75/76, His139/142, Asp189, Glu253 in RdMTO) that were proposed to mediate copper coordination at the putative active site of SELENBP1 [18].

A phylogenetic analysis, exploring the evolutionary relationship of SELENBP1 and RdMTO, resulted in a phylogenetic tree. It demonstrates the relations between vertebrate SeBPs, including human SELENBP1, and SeBPs from invertebrate, plant and prokaryotic model organisms and suggests that SELENBP1 and RdMTO are likely derived from a common bacterial ancestor (Figure 1B). This is further supported by a bioinformatics study published by Dervisi et al. who investigated the evolutionary origin of orthologous MTOs across archaea, bacteria, plants and animals. The authors proposed that SeBPs emerged from early prokaryotes, presumably as MTOs involved in the detoxification of harmful thiols in their environment [12]. The necessity to detoxify environmental MT is exemplified by the thermophilic and methanotrophic bacterium *Methylacidiphilum fumariolicum SolV*, which requires an MTO to counteract MT-derived growth inhibition [26]. Notably, the aforementioned amino acid residues that have been shown to be critical for MTO activity of either SELENBP1 (His73/74, His137/140, Asp189, Glu252) or RdMTO (Cys448) show conservation across the orthologous SeBPs from diverse species such as *C. elegans*, *Arabidopsis thaliana*, *Danio rerio*, *Mus musculus* and *Pongo abelii* (Figure S1), suggesting an evolutionarily conserved catalytic mechanism of enzymatic MT oxidation.

The 3D models of SELENBP1 (Figure 1C) and RdMTO (Figure 1D), as predicted by the AlphaFold database, reveal similar secondary and tertiary structural features of the two proteins: A complex arrangement of α-helices, β-sheets and loop regions converge into a globular β-propeller tertiary structure with a central cavity. The cysteine residues closest to the C-terminus of the proteins (Cys466 in SELENBP1 and Cys448 in RdMTO, respectively) are located within a loop facing the cavity at the inside of the barrel structure.

### 3.2. RdMTO Converts Methanethiol into H_2_S, H_2_O_2_ and HCHO in a Copper-Dependent Manner

**Figure 2:**
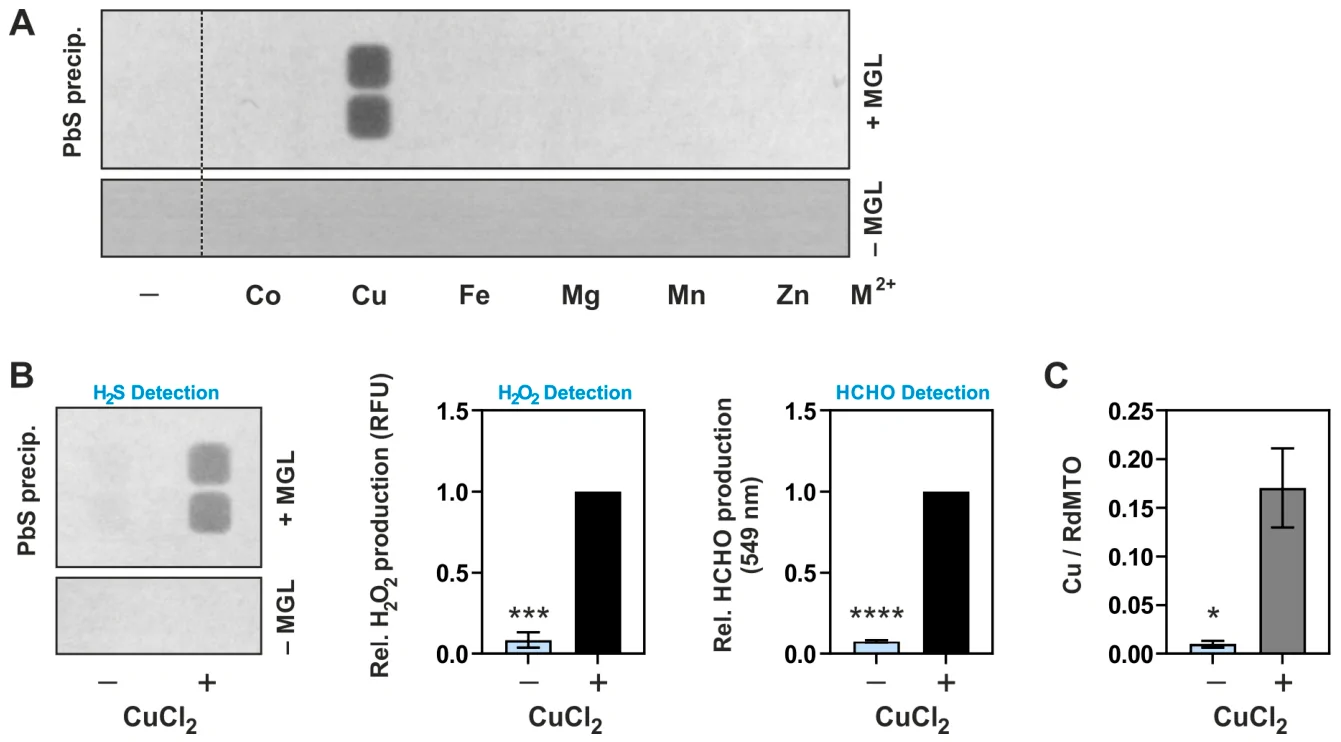
Copper-dependent conversion of MT to H_2_S, H_2_O_2_ and HCHO by RdMTO. (**A**) Isolated recombinant Strep-tagged RdMTO was supplemented with the given divalent cations (as chloride salts dissolved in HBS) to a final concentration of 15.38 µM (1:2 protein/cation). Levels of released volatile H_2_S were determined by the formation of PbS precipitates on lead acetate paper using a coupled enzyme assay. The assay was performed with (+MGL) or without (−MGL) the addition of MGL for in situ production of methanethiol. The image shows a lead acetate paper representative of three independent experiments. The images of the three biological replicates are provided in Figure S2. (**B**) Where indicated, the bacterial culture medium was supplemented with CuCl_2_ to a final concentration of 150 µM during overexpression of recombinant RdMTO. MTO activity of the purified RdMTO was determined by MT-derived H_2_S, H_2_O_2_ and HCHO production. H_2_S was detected as described above, while H_2_O_2_ was measured using a commercial peroxidase-dependent fluorescence-based assay kit. HCHO was determined spectrophotometrically using the Purpald reagent. The image of H_2_S detection by PbS precipitate formation on lead acetate paper is representative of three independent experiments. The images of all three biological replicates are shown in Figure S2. The graphs showing HCHO and H_2_O_2_ production represent means ± SD from three independent experiments; values obtained for RdMTO supplemented with CuCl_2_ were set to 1 and statistical analysis was carried out using a paired *t*-test (*** *p* < 0.001, **** *p* < 0.0001). (**C**) Recombinant RdMTO was produced in *E. coli* with or without CuCl_2_ supplementation of the bacterial culture medium, and copper content of the purified RdMTO was determined by TXRF. Data are means ± SD from three independent experiments; statistical analysis was carried out using a paired *t*-test (* *p* < 0.05).

The conservation of metal-binding motifs between SELENBP1 and RdMTO (Figure 1A), along with their common evolutionary origin (Figure 1B), suggested that copper may also serve as cofactor for the enzymatic activity of RdMTO. Thus, we first investigated the effect of divalent cations on formation of H_2_S by recombinant Strep-tagged RdMTO isolated from *E. coli*, applying a coupled in vitro assay that is based on in situ production of volatile MT [25]. Similarly to SELENBP1 [18], MT-derived H_2_S formation was strongly elevated if RdMTO was pretreated with Cu(II)-ions. In contrast, prior addition of Co^2+^, Fe^2+^, Mg^2+^, Mn^2+^ or Zn^2+^ to RdMTO did not result in MT-derived H_2_S formation (Figure 2A). To further substantiate Cu dependency of RdMTO activity, the two other products of SELENBP1-catalyzed MT oxidation [11], H_2_O_2_ and HCHO, were detected in parallel with H_2_S in a second set of experiments: Both H_2_O_2_ and HCHO were barely detectable if the recombinant RdMTO was not provided with Cu^2+^. On the other hand, all three products, H_2_S, H_2_O_2_ and HCHO, were generated in the coupled enzyme assay if the applied RdMTO was isolated from *E. coli* grown in culture medium supplemented with CuCl_2_ (Figure 2B). The copper content of recombinant RdMTO isolated from non-supplemented *E. coli* bacteria was very low, with an average copper-to-RdMTO protein ratio of approximately 0.01, and this ratio increased 17-fold in response to supplementation of the bacterial culture medium with CuCl_2_ during protein overexpression (Figure 2C).

In summary, RdMTO is capable of converting MT to H_2_S, H_2_O_2_ and HCHO, provided that copper is available as cofactor. RdMTO is now the fourth known SeBP ortholog with demonstrated Cu-dependent MTO activity, next to human SELENBP1, SEMO-1 from *C. elegans* and another bacterial SeBP ortholog from *Hyphomicrobium* sp. [14,18].

In apparent contrast to our findings, Cao et al. reported that RdMTO does not produce H_2_S and H_2_O_2_, while proposing S^0^, HCHO and H_2_O as the final products of RdMTO-catalyzed MT oxidation [13]. It should be noted, however, that the authors neither explored the effect of Cu on the enzymatic activity of their recombinant His-tagged protein nor its Cu content. Moreover, His-tags bind Cu(II) ions with high efficiency [27], which might have further limited the availability of Cu for coordination with amino acids in the active site of RdMTO in their study. Although it appears conceivable that availability and binding of copper might shape not only the extent but also the mode of MT conversion by RdMTO (as well as by other SeBPs), this is unlikely to fully explain the different outcome of the two studies. We found that formation of HCHO by Cu-supplemented RdMTO was coupled to formation of H_2_S and H_2_O_2_, whereas the three breakdown metabolites of MT were not or barely detectable if Cu-deficient RdMTO was applied (Figure 2B). By contrast, RdMTO-catalyzed conversion of MT to HCHO was coupled to formation of S^0^ in the study by Cao et al. [13]. Nevertheless, a recent study has indeed revealed the first example of a functional switch in enzymatic MT conversion that had occurred during the evolution of malodorous plants. By exchange of only three amino acids at their putative active site, including one that was previously demonstrated by us to participate in Cu binding of SELENBP1, the enzymatic activity of SeBPs from some *Asarum*, *Symplocarpus* and *Eurya* species had shifted from that of an MTO to a disulfide synthase activity. MT is not converted into H_2_S anymore, but rather into two other VSCs, dimethyl disulfide (DMDS) and dimethyl trisulfide (DMTS) [15]. Binding of other divalent metal ions such as zinc may also provide a potential way of shifting the catalytic activity of enzymes. Such an example is found in the tyrosinase protein family, which comprises in animals tyrosinase and the tyrosinase-related proteins Trp1 and Trp2, originating from gene triplication. Tyrosinase catalyzes the copper-dependent oxidation of tyrosine to dopaquinone, whereas Trp2 is a zinc-dependent dopachrome tautomerase [28]. Interestingly, SELENBP1 has been reported to bind both copper and zinc [29] but it remains to be elucidated whether binding of zinc is increased under copper-deficient conditions and may alter the MTO activity towards S^0^ formation.

### 3.3. RdMTO Accepts Structurally Related Alkyl Thiols as Substrates

Several short-chain alkyl thiols other than MT, such as ethanethiol (ET) and propanethiol (PT), can serve as substrates of SELENBP1 [18]. To test whether this applies to RdMTO as well, we assessed the formation of H_2_S, H_2_O_2_ and HCHO from MT, in comparison to ET and PT, applying recombinant RdMTO produced in *E. coli* supplemented with CuCl_2_. The highly volatile thiols MT and ET were generated in situ through MGL-catalyzed degradation of their precursors methionine and ethionine, respectively. The less volatile PT was added directly to the reaction mix.

**Figure 3:**
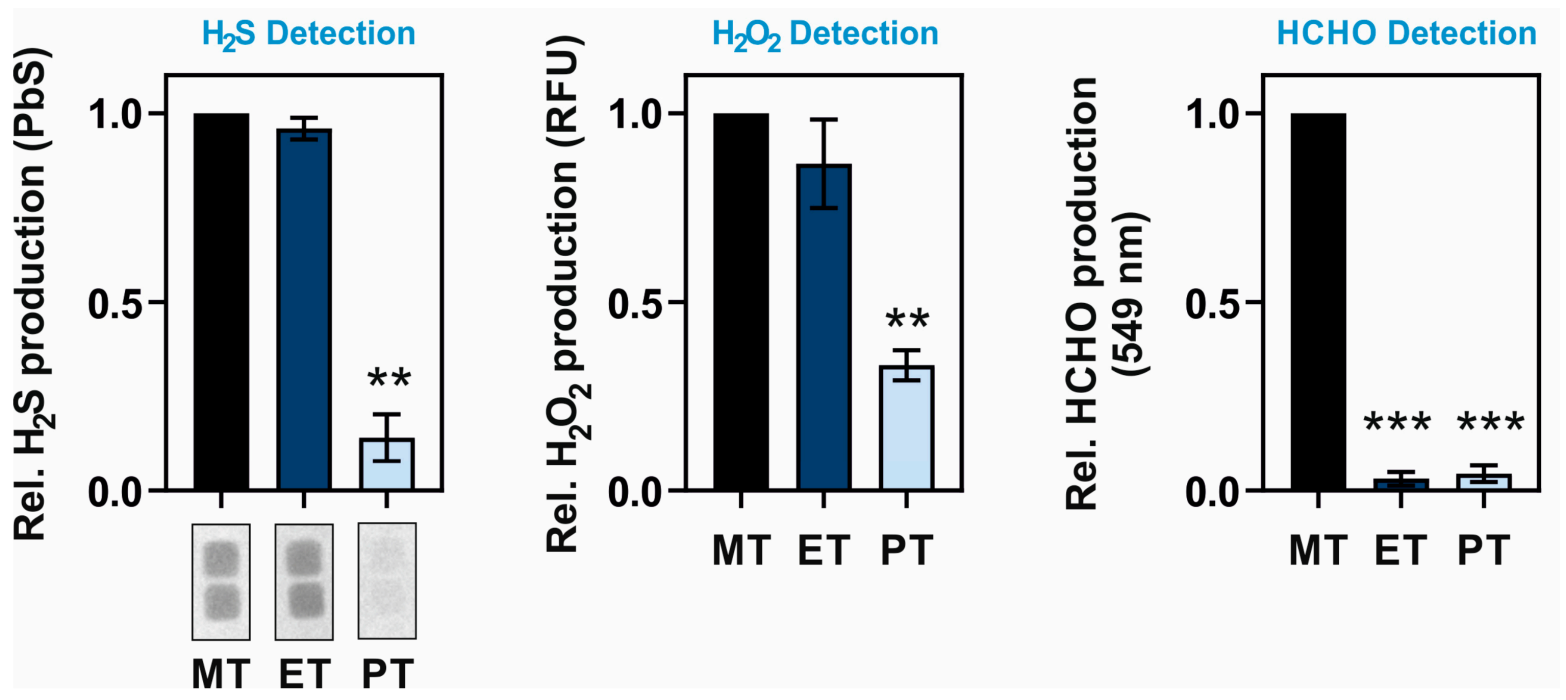
Alkyl thiol substrates of RdMTO. Recombinant Strep-tagged RdMTO was produced in *E. coli* grown in culture medium supplemented with CuCl_2_ to a final concentration of 150 µM and purified using Strep-tag affinity chromatography. MTO activity was tested by determining H_2_S, H_2_O_2_ and HCHO production with MT, ET or PT as substrates. MT and ET were enzymatically generated via MGL-catalyzed degradation of their precursor amino acids L-methionine and DL-ethionine, respectively, while PT was added directly to the reaction mix. Data represent means ± SD from three independent experiments, normalized to values obtained with MT as substrate; statistical analysis was carried out using one-way ANOVA with Dunnett’s multiple comparisons test (** *p* < 0.01, *** *p* < 0.001). The images of all three biological replicates of H_2_S detection are provided in Figure S3.

RdMTO was capable of producing H_2_S and H_2_O_2_ from each of the three alkyl thiols. Similar levels of both H_2_S and H_2_O_2_ were detected with MT and ET as substrates, whereas PT was converted less efficiently (Figure 3). This pattern closely resembles the observations reported for recombinant SELENBP1 [18]. Moreover, purified MTOs from *Thiobacillus thioparus* and *Hyphomicrobium* bacteria were previously shown to oxidize MT and ET equally well [30,31]. In contrast to H_2_S and H_2_O_2_, HCHO formation was detected only with MT as substrate (Figure 3), which was expected, as the oxidation of ET and PT results in formation of acetaldehyde and propionaldehyde, respectively.

While the products of MTO-catalyzed alkyl thiol oxidation fulfill physiological roles, e.g., as signaling molecules and as metabolites in carbon and sulfur metabolism, MT and ET at high concentrations elicit cytotoxic effects in humans, e.g., through interaction with the mitochondrial respiratory chain by inhibiting cytochrome *c* oxidase [32,33]. Similarly, MTOs in bacteria may be primarily required for detoxification of harmful thiols, but they may also participate in modulating cellular redox homeostasis and in providing metabolites for anabolic pathways. In this regard, radiotracer experiments conducted with marine microorganisms have demonstrated that, following the breakdown of DMSP, trace quantities of MT are absorbed and subsequently utilized as sulfur source by marine bacterioplankton [21].

### 3.4. A Conserved Cysteine Residue Close to the C-Terminus of Both RdMTO and SELENBP1 Is Relevant but Not Essential for Their MTO Activity

Three conserved cysteine residues were previously identified in *Rhodobacteraceae* MTOs [13]; interestingly, they are conserved in human SELENBP1 as well. Cys82, Cys143 and Cys448 in RdMTO correspond to Cys80, Cys141 and Cys466 in SELENBP1 (Figure 1A). Mutation of each of these three cysteine residues in RdMTO reportedly resulted in loss of MTO activity, as assessed by detection of MT-derived S^0^ formation [13]. By contrast, we had previously investigated the role of cysteine residues in SELENBP1 and found that mutation of two of the corresponding cysteine residues, Cys80 and Cys141, did not suppress MTO activity. MT-derived H_2_S and H_2_O_2_ formation by these mutants was rather slightly (by trend) increased, as compared to the wild-type protein [18].

**Figure 4:**
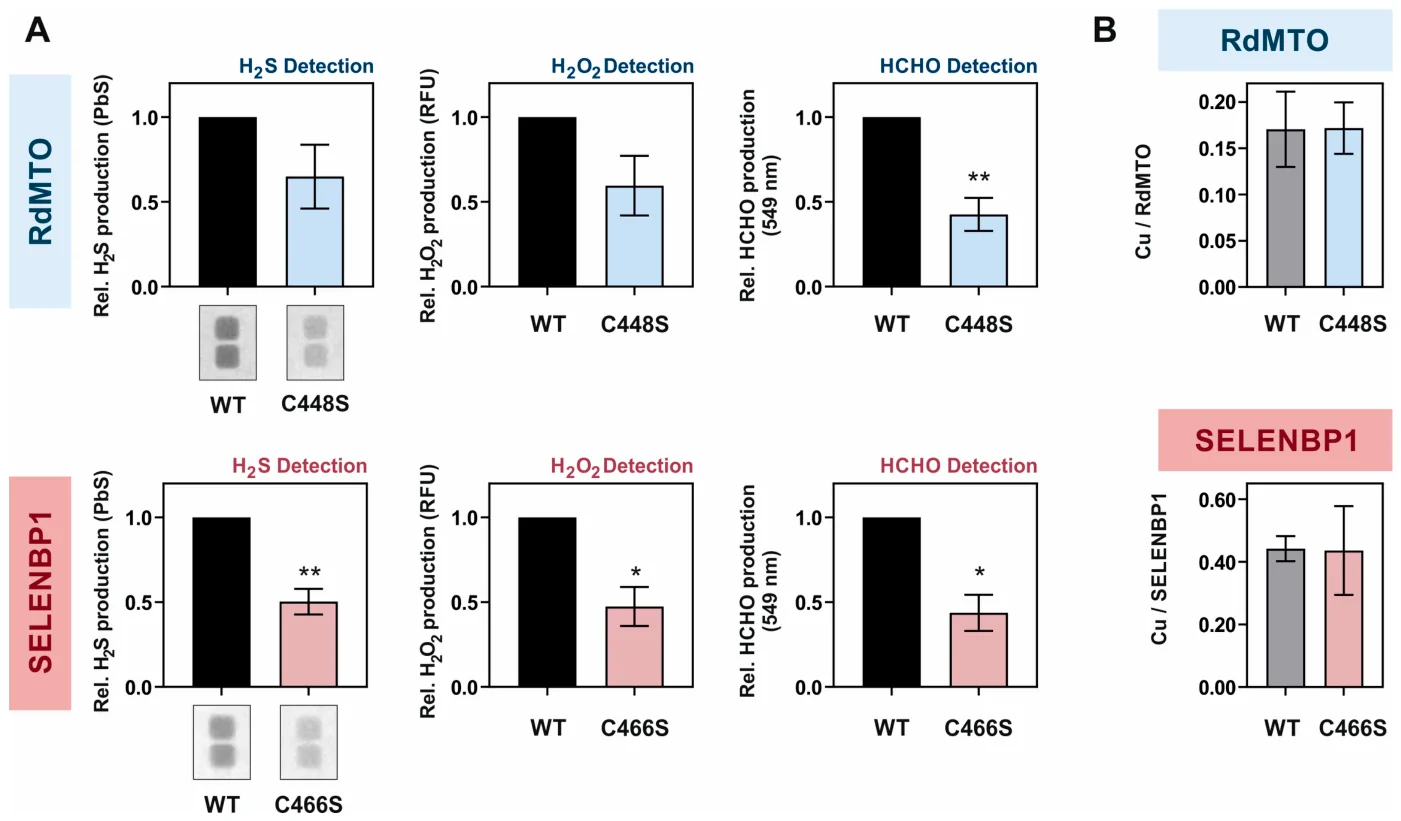
Role of Cys448 in RdMTO and Cys466 in SELENBP1 in MTO activity and copper binding. RdMTO (C448S) and SELENBP1 (C466S) mutants were generated through site-directed in vitro mutagenesis. Wild-type (WT) and mutant proteins were produced in *E. coli* as recombinant Strep-tagged proteins, with copper supplementation of the bacterial culture medium as described in Figure 2B and Figure 3. (**A**) MTO activity of WT and mutant proteins was assessed from MT-derived H_2_S, H_2_O_2_ and HCHO release. Data are means ± SD from three independent experiments, normalized to WT. The images of the three biological replicates of H_2_S detection are provided in Figure S4. (**B**) Copper content of the recombinant RdMTO and SELENBP1 WT and mutant proteins was determined by TXRF. Data are means ± SD from three independent experiments; statistical analysis was carried out using a paired *t*-test (* *p* < 0.05, ** *p* < 0.01).

To address these differences, we evaluated the functional relevance of the cysteine residue closest to the C-terminus of the proteins for their copper-dependent MTO activity. The respective cysteine-deficient mutants (RdMTO Cys448Ser and SELENBP1 Cys466Ser) were generated and produced as recombinant proteins in *E. coli* supplemented with CuCl_2_. The purified Strep-tagged proteins were then assessed for MT-derived H_2_S, H_2_O_2_ and HCHO production. Both mutant proteins indeed showed decreased (by up to 55%, as compared to the wild-type proteins), but not abrogated, formation of the three products of MT oxidation (Figure 4A). This indicates an important, but non-essential, role of the C-terminal cysteine residue for their MTO activity.

Cys466 thus appears to be the only cysteine residue in SELENBP1 whose mutation results in marked lowering of its MTO activity. Moreover, this cysteine residue is highly conserved among orthologous SeBPs/MTOs across multiple species (Figure S1), suggesting a structural and/or functional role. In 2011, Raucci et al. generated a 3D model of SELENBP1 with the use of a bacterial SeBP ortholog from *Sulfolobus tokodaii* as template, proposing the stabilization of the tertiary structure of SELENBP1 through two intramolecular disulfide bridges (Cys80/Cys141 and Cys83/Cys466) [34]. Disulfide bonds have an average length of 2.05 Å, and therefore, 2.5 Å is usually considered the threshold value for the computational prediction of disulfides within proteins [35,36]. Using up-to-date 3D models of SELENBP1 and RdMTO provided by the AlphaFold database as well as the PyMOL program for the calculation of spatial distances between thiol groups in cysteine residues of proteins, we found that Cys80/Cys141 and Cys82/Cys143 may form disulfide bridges in SELENBP1 and RdMTO (Figure 1C,D), respectively, as the predicted distances between them are within the range of 2.5 Å. By contrast, the calculated distances for Cys83/Cys466 in SELENBP1 and for Cys85/Cys448 in RdMTO were found to be 18.7 Å and 18.4 Å, respectively, arguing against the formation of an intramolecular disulfide bridge with the participation of the cysteine residue closest to the C-terminus of the proteins.

It was previously proposed that Cys448 in RdMTO may directly bind MT, implying a catalytic mechanism based on the formation of a Cys448-S-S-CH_3_ intermediate with subsequent electrophilic attack by oxygen to cleave the C-S bond [13]. MT is indeed known for its potential to form disulfide bonds with cysteine residues in proteins [37]. The computational 3D protein structures show that both Cys466 in SELENBP1 (Figure 1C) and Cys448 in RdMTO (Figure 1D) are located within flexible loops facing the internal cavity of the proteins, thus allowing for an interaction with MT. However, if these residues were essential components of the active site of the enzymes, their mutation would be expected to abolish MT conversion completely. Since only a partial loss of MT-derived H_2_S, H_2_O_2_ and HCHO production catalyzed by the mutant proteins was observed here (Figure 4A), it appears unlikely that the C-terminal cysteine residues are directly involved in MT binding.

Copper ions predominantly coordinate into proteins via amino acid residues with nitrogen and sulfur donor atoms such as histidine, cysteine and methionine [38]. Recently, we proposed His137, His140, Asp189 and Glu252 as potential copper ligands within the putative active site of SELENBP1, as MTO activity and copper-to-protein ratio of mutant proteins with a single exchange of each of these amino acids were abrogated and significantly lowered, respectively, compared to the wild-type protein [18]. Further evidence for a cysteine-independent coordination of copper stems from structural studies on the recombinant MTO from *Hyphomicrobium* sp. Electron paramagnetic resonance spectroscopy and quick extended X-ray absorption fine structure analysis suggested copper coordination via four nitrogen ligands from histidine residues [14]. Nevertheless, a role of the C-terminal cysteine residue in copper binding of RdMTO and SELENBP1 cannot be entirely ruled out; this would, in part, explain the observed attenuation of enzymatic activity by mutation of these cysteines. Therefore, we compared the copper content of recombinant RdMTO and SELENBP1 wild-type and mutant proteins. The average copper-to-protein ratio was 0.17 for both RdMTO and its Cys448Ser mutant, while both SELENBP1 and its Cys466Ser mutant showed an average copper-to-protein ratio of 0.4 (Figure 4B). Copper binding, therefore, was not altered by mutation of Cys448 in RdMTO or Cys466 in SELENBP1, suggesting that these cysteine residues are unlikely to be directly involved in copper coordination or exert an indirect structural effect on the accessibility of copper binding amino acid residues. Strikingly, the copper-to-protein ratios were rather low for both SELENBP1 and RdMTO, which might be due to only partial copper occupancy of their catalytic site and thus the presence of residual apo-proteins. It should be noted that both proteins were overexpressed in *E. coli* bacteria as recombinant proteins, which might have affected their three-dimensional folding and thus the accessibility of their active sites for copper ions. Moreover, the chaperones for delivery of copper to newly synthesized proteins differ between different species. Thus, future in vivo experiments using cultured cells, animal models and *R. denitrificans* will be helpful to obtain further support for the role of copper as required MTO cofactor and to clarify the catalytic mechanism of MTOs.

Taken together, these data suggest that the C-terminal cysteine residue of both RdMTO and SELENBP1 is neither involved in copper binding nor in formation of intramolecular disulfide bridges. Regarding the reason for the lowered MTO activity of the mutant proteins, we can only speculate at this point. The contribution of the C-terminal cysteine residue may relate to influencing the local electrochemical environment or facilitating substrate access to the catalytic site.

## 4. Conclusions

Several orthologous SeBPs from diverse species have been reported to exhibit MTO activity [11,13,14,15,16,17]. In this study, we show that the recently identified RdMTO from the marine bacterium *R. denitrificans* and human SELENBP1 are orthologous SeBPs that are presumably derived from a common bacterial ancestor. Both proteins catalyze the conversion of MT and structurally related alkyl thiols to H_2_S, H_2_O_2_ and HCHO/RCHO, respectively, in a copper-dependent manner. Notably, several amino acid residues proposed to coordinate copper as well as a cysteine residue close to the C-terminus that appears to be important for full MTO activity are conserved in both enzymes. Our data support the scheme of enzymatic MT oxidation originally postulated in 2018 in the landmark study by Pol et al. [11] and suggest that the catalytic mechanism of MTOs is evolutionarily conserved from bacteria to humans. To obtain further insights, future studies may address kinetic parameters such as the Michaelis–Menten constant and the turnover number of orthologous SeBPs/MTOs from different species as well as the role of conserved amino acid residues in copper coordination, substrate binding and product formation, preferably through applying a recently established novel fluorometric MTO assay [39].

The capability to oxidize MT appears to be an early invention of prokaryotes, presumably in order to cope with toxic thiols in their environment. In this regard, a recently published large bioinformatics study came to the conclusion that oxidoreductases, and, in particular, metal-binding enzymes, remained highly conserved during evolution, with emphasis on their catalytic sites and the stability of once-established metabolic networks [40]; SeBPs/MTOs appear to be a perfect example supporting this hypothesis.

## Figures and Tables

**Table 1:** Sequences of primers used for cloning of RdMTO (KEGG-ID: RD1_2378).

Primer Name	Sequence (5′ → 3′)
CP1_RdMTO_F	ggaccgcctgatcacaattc
CP1_RdMTO_R	cgcggtttcaaaggcaaaag
CP2_RdMTO_NdeI_F	gcccatatgacagagacaaacggaagc
CP2_RdMTO_Eco47III_R	aacagcgctcccatcaatccagatgtcggatgagca

CP (cloning primer).

**Table 2:** Sequences of primers used for site-directed in vitro mutagenesis of RdMTO (KEGG-ID: RD1_2378) and human SELENBP1 (Gene-ID: NM_003944).

Primer Name	Sequence (5′ → 3′)
MP_RdMTO_C448S_F	ggcggtgatt**c**ctcatccgacatctg
MP_RdMTO_C448S_R	cagatgtcggatgag**g**aatcaccgcc
MP_SELENBP1_C466S_F	accctgggggcgatt**c**tagctctgacatctgg
MP_SELENBP1_C466S_R	ccagatgtcagagcta**g**aatcgcccccagggt

MP (mutagenesis primer; bases altered through in vitro mutagenesis are highlighted in bold and underlined).

## Data Availability

The original contributions presented in the study are included in the article, further inquiries can be directed to the corresponding authors.

## Supplementary Materials

The following supporting information can be downloaded at https://www.mdpi.com/article/10.3390/antiox15091148/s1, Figure S1: Multiple protein sequence alignment of orthologous SeBPs from diverse organisms; Figure S2: Copper-dependent conversion of MT to H_2_S by RdMTO; Figure S3: RdMTO-mediated conversion of the alkyl thiols MT, ET and PT to H_2_S; Figure S4: Role of Cys466 in SELENBP1 and Cys448 in RdMTO for MT-derived H_2_S production.

## Abbreviations

The following abbreviations are used in this manuscript:

*C. elegans*	*Caenorhabditis elegans*
DMDS	Dimethyl disulfide
DMSP	Dimethylsulfoniopropionate
DMTS	dimethyl trisulfide
*E. coli*	*Escherichia coli*
ET	Ethanethiol
HBS	HEPES-buffered saline
HCHO	Formaldehyde
HRP	Horseradish peroxidase
H_2_O_2_	Hydrogen peroxide
H_2_S	Hydrogen sulfide
KEGG	Kyoto Encyclopedia of Genomes and Genes
MGL	Methionine γ-lyase
MT	Methanethiol
MTO	Methanethiol oxidase
PT	Propanethiol
*R. denitrificans*	*Roseobacter denitrificans*
S^0^	Sulfane sulfur
SeBP	Selenium-binding protein
SELENBP1	Selenium-binding protein 1
SEMO-1	SELENBP1 ortholog with MTO activity
SNP	Single nucleotide polymorphism
TXRF	Total reflection X-ray fluorescence
VSC	Volatile sulfur compound
