# Supplementary material for: Evolutionary Conservation of the Copper-Dependent Thiol Oxidase Activity of Selenium-Binding Proteins
Source: Antioxidants (Basel). 2026 Sep 10;15(9):1148. doi: 10.3390/antiox15091148 (PMC13603461; doi:10.3390/antiox15091148)
Supplement: Supplementary file 1 [file antioxidants-15-01148-s001.zip › antioxidants-4512437-supplementary.pdf]

Schlemminger et al., SUPPLEMENTARY FIGURES

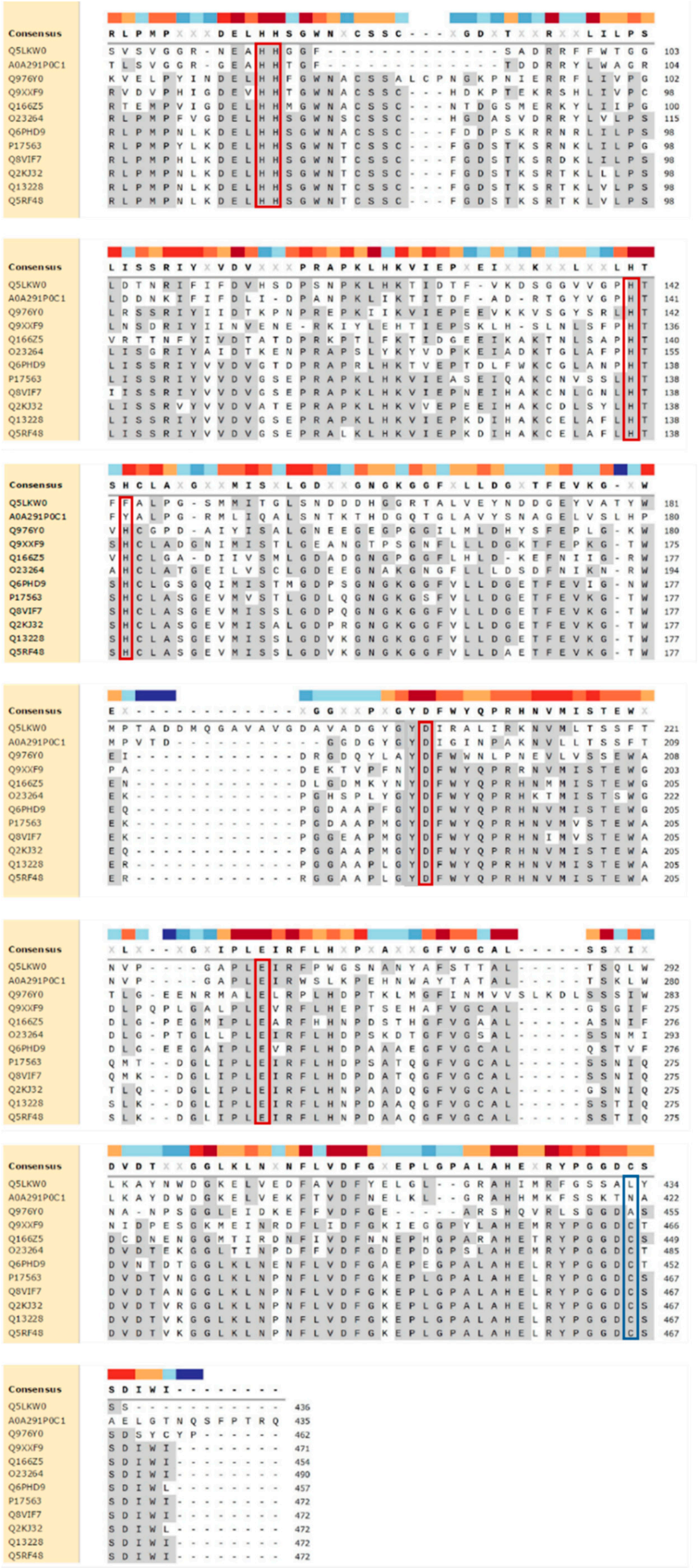

Figure S1. Multiple protein sequence alignment of orthologous SeBPs from diverse organisms. Protein sequences were obtained from the UniProt database (https://www.uniprot.org/). Conserved amino acid residues are shown in grey, with postulated copper ligands framed in red and the C-terminal cysteine residue highlighted in blue. MTO UniProt IDs - organism: Q5LKW0 - *Ruegeria pomeroyi;* A0A291P0C1 - *Hyphomicrobium sp;* Q976Y0 - *Sulfolobus tokodaii;* Q9XXF9 - *Caenorhabditis elegans;* Q166Z5 - *Roseobacter denitrificans;* O23264 - *Arabidopsis thaliana;* Q6PHD9 - *Danio rerio;* P17563 - *Mus musculus;* Q8VIF7 - *Rattus norvegicus;* Q2KJ32 - *Bos taurus;* Q13228 - *Homo sapiens;* Q5RF48 - *Pongo abelii*

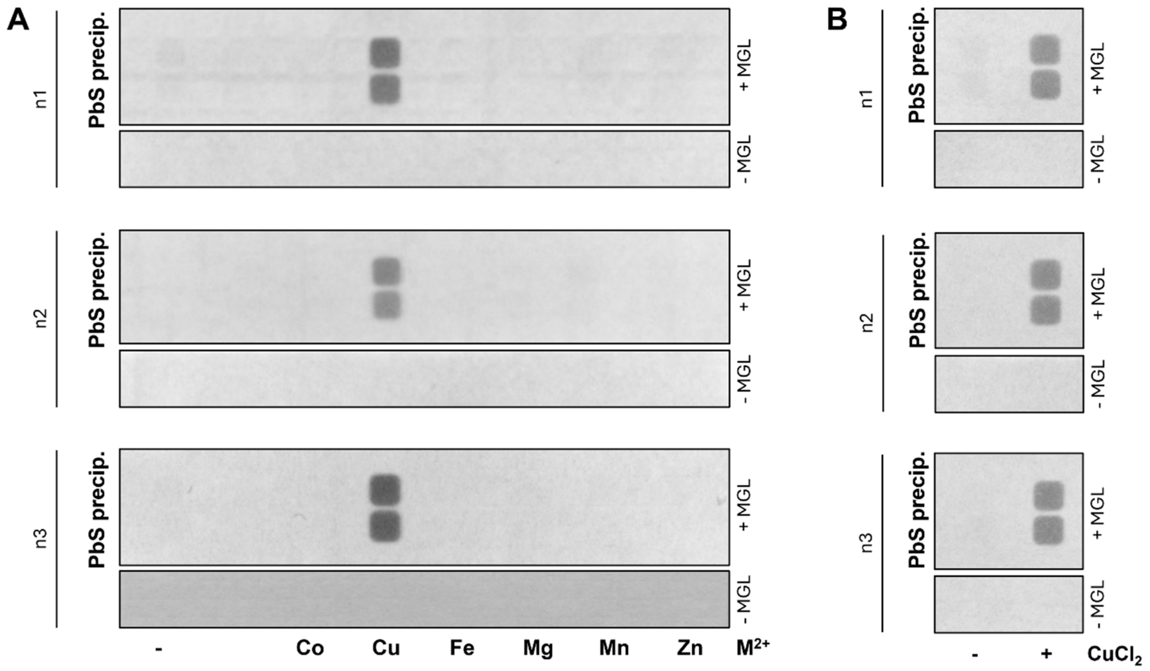

Figure S2. Copper-dependent conversion of MT to H_2S by RdMTO. (A) Isolated recombinant Strep-tagged RdMTO was supplemented with the given divalent cations (as chloride salts dissolved in HBS) to a final concentration of 15.38 µM (1:2 protein/cation). Levels of released volatile H_2S were determined by the formation of PbS precipitates on lead acetate paper using a coupled enzymatic assay. The assay was performed with (+ MGL) or without (- MGL) the addition of MGL for *in situ*-production of MT. (B) The bacterial culture medium was supplemented with $CuCl_2$ to a final concentration of 150 µM during overexpression of recombinant RdMTO, where indicated. MT-derived H_2S production was determined using the coupled enzymatic assay as described above.

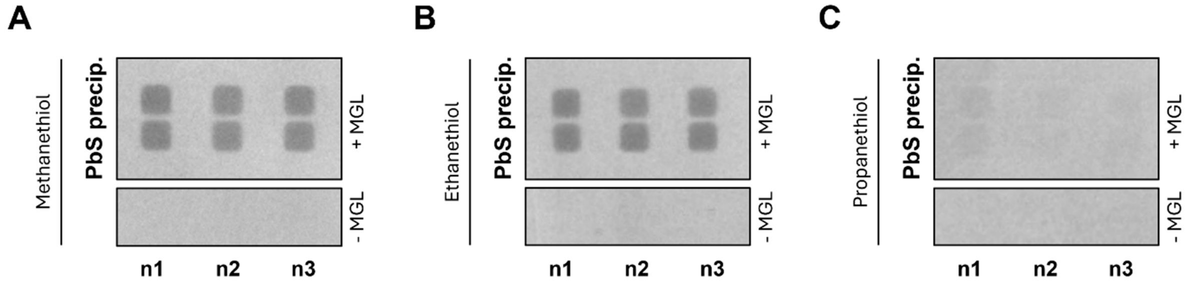

Figure S3. RdMTO-mediated conversion of the alkyl thiols MT, ET and PT to H_2S. Recombinant Strep-tagged RdMTO was produced in *E. coli* supplemented with $CuCl_2$ to a final concentration of 150 µM during overexpression and purified using Strep-tag affinity chromatography. H_2S production was determined using the coupled enzymatic assay using MT (A), ET (B) or PT (C) as substrates. MT and ET were enzymatically generated via MGL-catalyzed degradation of their precursor amino acids methionine and ethionine, respectively, while PT was added directly to the reaction mix.

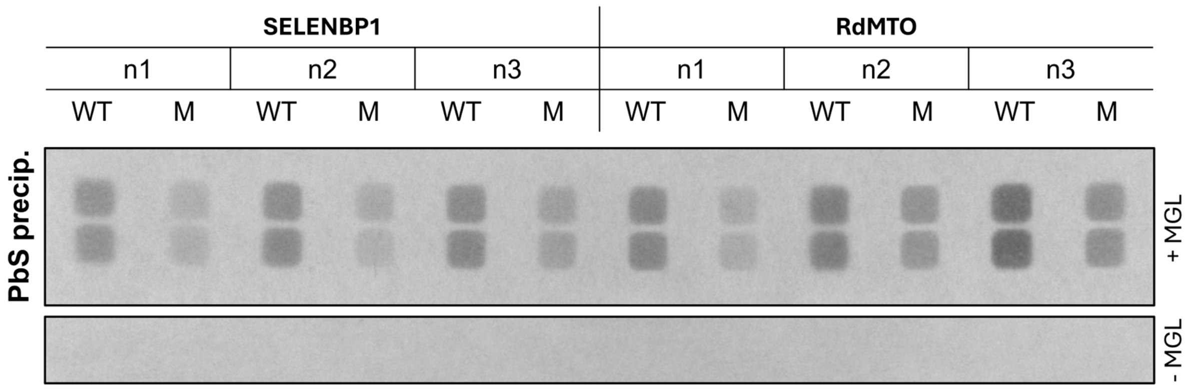

Figure S4. Role of Cys466 in SELENBP1 and Cys448 in RdMTO for MT-derived H_2S production. SELENBP1 (C466S) and RdMTO (C448S) mutants were generated through site-directed *in vitro* mutagenesis. Wild-type (WT) and mutant proteins (M) were produced in *E. coli* as recombinant Strep-tagged proteins, with copper supplementation of the bacterial culture medium as described in Fig. S3. MT-derived H_2S production was assessed as described in Fig. S2.
